# Supplementary material for: Machine Learning Reveals Lipidome Remodeling Dynamics in a Mouse Model of Ovarian Cancer
Source: J Proteome Res. 2023 May 23;22(6):2092–108. doi: 10.1021/acs.jproteome.3c00226 (PMC10243112; doi:10.1021/acs.jproteome.3c00226)
Supplement: Supplementary file 1 — pr3c00226_si_001.pdf [file pr3c00226_si_001.pdf]

Supporting information for

Machine Learning Reveals Lipidome Remodeling Dynamics in a Mouse Model of Ovarian Cancer.

Olatomiwa O. Bifarin[1,†], Samyukta Sah[1,†], David A. Gaul[1,4], Samuel G. Moore[4], Ruihong Chen[2], Murugesan Palaniappan[2,3], Jaeyeon Kim[5], Martin M. Matzuk[2,3], Facundo M. Fernández[1,4]*

[1]School of Chemistry and Biochemistry, Georgia Institute of Technology, Atlanta, Georgia 30332, United States.

[2]Department of Pathology & Immunology, Baylor College of Medicine, Houston, TX 77030, United States.

[3]Center for Drug Discovery, Department of Pathology & Immunology, Baylor College of Medicine, Houston, TX 77030, United States.

[4]Petit Institute of Bioengineering and Bioscience, Georgia Institute of Technology, Atlanta, Georgia 30332, United States.

[5]Department of Biochemistry and Molecular Biology, Indiana University School of Medicine, Indiana University Melvin and Bren Simon Comprehensive Cancer Center, Indianapolis, Indiana, 46202, United States.

*Corresponding author. Email: facundo.fernandez@chemistry.gatech.edu (F.M.F)

† These authors contributed equally to this work.

Table of Contents

Figure S1: Survival analysis comparison of DKO and DKO control mice.

Figure S2: DKO and DKO control mice comparison via unsupervised learning methods.

Figure S3: Permutation test plots for validating the best-performing ML models used for discriminating DKO from DKO control mice.

Figure S4: Restricted Mean Survival Times (RMST) plots for all prognostic lipid candidates.

Table S1: Eighty-seven statistically significant ($q < 0.05$) lipids for the DKO vs. DKO control comparison, all time points combined.

Table S2: Statistically significant lipid features for the comparison between DKO and DKO control mice that were present in at least three lifetime stages.

Table S3: Lipids selected *via* machine learning for each percentage lifetime stage.

Table S4: Machine learning results for DKO classification.

Table S5: Composition of stable isotope-labeled chemical standards mixture used in UHPLC-MS;

Table S6: Chromatographic gradient for RP UHPLC-MS method (PDF)

Table S7: MS parameters used for RP UHPLC-MS.

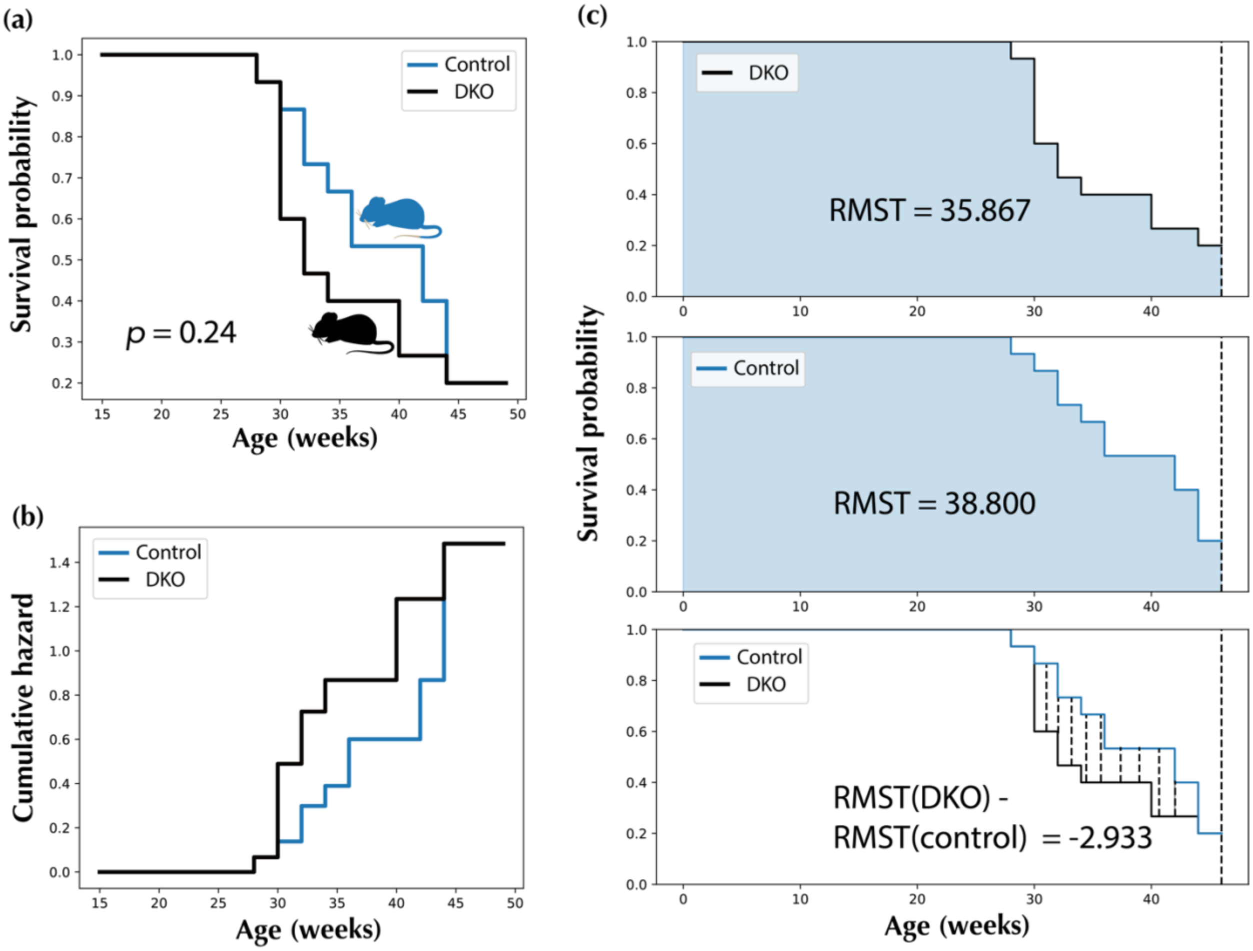

Figure S1. Survival analysis comparison of DKO and DKO control mice. (a) Kaplan-Meier survival curve estimate, DKO *vs.* DKO control mice. (b), Nelson-Aalen hazard curve estimate, DKO *vs.* DKO control mice. (c), Restricted Mean Survival Times (RMST), DKO *vs.* DKO control mice.

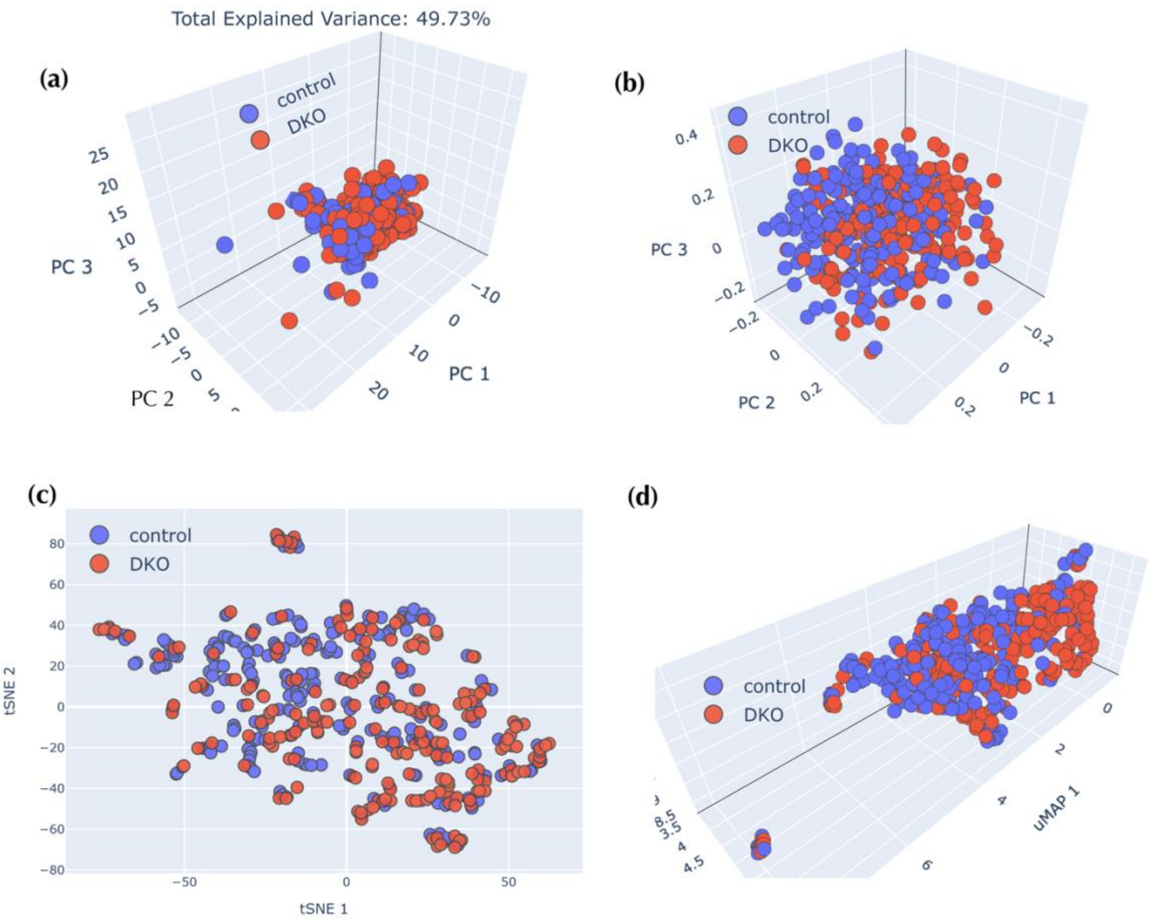

Figure S2. DKO and DKO control mice comparison *via* unsupervised learning methods. (a), PCA score plot. (b), Kernel PCA score plot. (c), tSNE score plot. (d), UMAP score plot. Eighty-seven statistically significant lipid abundances were used for unsupervised learning, and all-time points were combined.

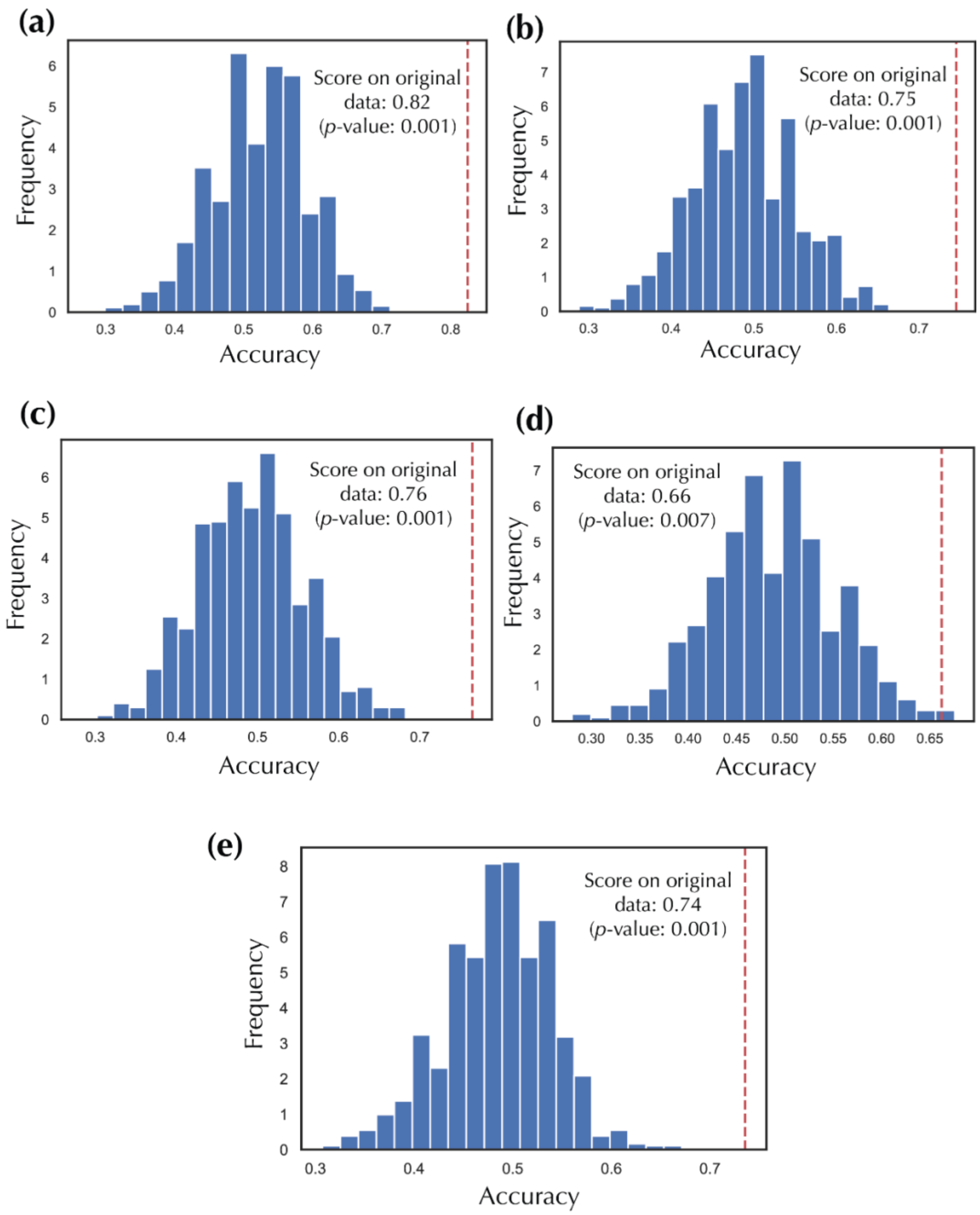

Figure S3. Permutation test plots for validating the best-performing ML models used for discriminating DKO from DKO control mice. (a) lifetime stage I, voting ensemble classifier, **(b)** lifetime stage II, RF classifier, **(c)** lifetime stage III, voting ensemble classifier, **(d)** lifetime stage IV, RF classifier, **(e)** lifetime stage V, SVM classifier. The score on the original data is the average mean accuracy score of the entire dataset under five-fold cross-validated conditions. *P*-values are calculated by the ratio of permutations in which the accuracy score of the permuted dataset exceeds the accuracy achieved with the unpermuted dataset.

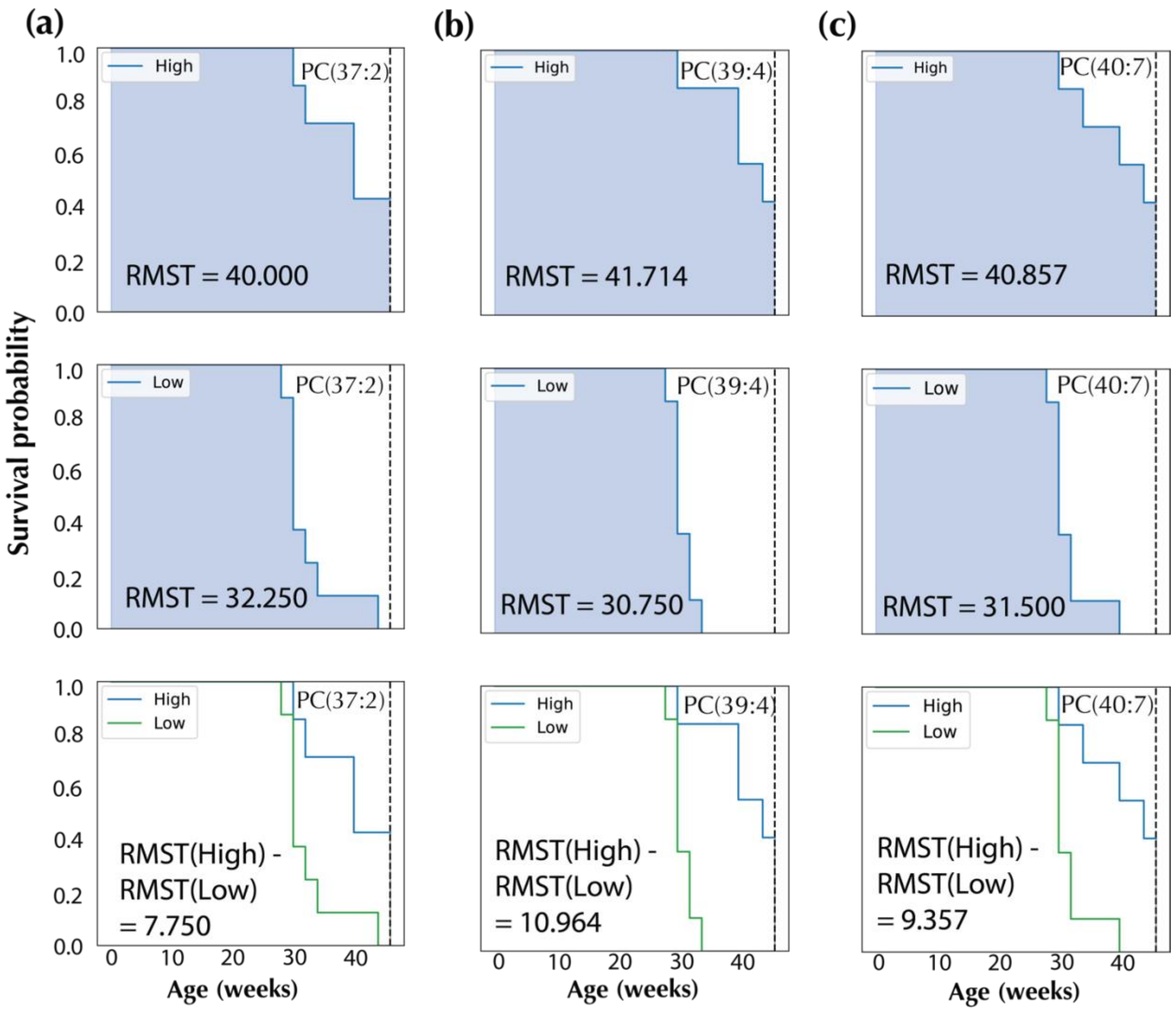

Figure S4. Restricted Mean Survival Times (RMST) plots for all prognostic lipid candidates. (a), PC(37:2). (b), PC(39:4). (c), PC(40:7).

Table S1. Eighty-seven statistically significant (*q* < 0.05) lipids for the DKO *vs*. DKO control comparison, all time points combined. DG: Diacylglycerols, TG: Triacylglycerols, FA: Fatty acids, HexCer: Hexosylceramides, LPC: Lysophosphatidylcholines, LPE: Lysophosphatidylethanolamines, PC: Phosphatidylcholines, PC-O: Ether phosphatidylcholines, PE: Phosphatidylethanolamines, PE-O: Ether phosphatidylethanolamines, PI: Phosphatidylinositols, Cer: Ceramides, and SM: Sphingomyelins Confidence level for metabolite annotation was assigned based on the following criteria: 1) exact mass, isotopic pattern, retention time, and MS/MS spectrum of standard matched to the feature. 2) exact mass, isotopic pattern, and MS/MS spectrum matched with in-house MS/MS library or literature spectra, or fragmentation ions observed are consistent with proposed structure. 3) tentative ID assignment based on elemental formula match with literature. 4) unknowns.

ID	Retention Time [min]	Lipids	Adduct	Confidence Level
24	1.54	FA(14:1)	$[M-H]^-$	2
1472	4.67	PE(O-34:3)	$[M-H]^-$	2
1111	4.69	Cer(d33:1)	$[M+CH_3COOH-H]^-$	2
966	4.70	Cer(d34:1)	$[M-H]^-$	2
1149	4.72	Cer(d34:1)	$[M+CH_3COOH-H]^-$	2
1217	6.83	Cer(d40:1)	$[M-H]^-$	2
1454	6.64	Cer(d41:2)	$[M+CH_3COOH-H]^-$	2
1297	6.79	Cer(d42:2)	$[M-H]^-$	2
1290	6.46	Cer(d42:3)	$[M-H]^-$	2
1504	6.46	Cer(d42:3)	$[M+CH_3COOH-H]^-$	2
111	2.37	FA(18:1)	$[M-H]^-$	2
1473	4.33	HexCer(d34:1)	$[M-H]^-$	2
1761	4.09	HexCer(d34:1)	$[M+CH_3COOH-H]^-$	2
1762	4.32	HexCer(d34:1)	$[M+CH_3COOH-H]^-$	2
2078	6.83	HexCer(d42:1)	$[M+CH_3COOH-H]^-$	2
2532	6.83	HexCer(d42:1)	$[M-H]^-$	2
2065	6.41	HexCer(d42:2)	$[M-H]^-$	2

2522	6.41	HexCer(d42:2)	$[M+CH_3COOH-H]^-$	2
4260	6.51	HexCer(d42:2)	$[M-H]^-$	2
2415	6.41	HexCer(d42:2)	$[M+H_2CO_2-H]^-$	2
257	2.03	FA(20:4-2OH)	$[M-H]^-$	2
52	2.02	FA(16:1)	$[M-H]^-$	2
1870	4.26	PC(16:0_16:0)	$[M+H_2CO_2-H]^-$	2
2050	5.45	PC(16:0_18:0)	$[M+H_2CO_2-H]^-$	2
2110	3.76	PC(16:0_18:2)	$[M+CH_3COOH-H]^-$	2
1679	4.14	PC(16:0_18:2)	$[M+H_2CO_2-H]^-$	2
1751	4.51	PC(16:0_18:2)	$[M+H_2CO_2-H]^-$	2
2265	5.90	PC(18:0_18:0)	$[M+H_2CO_2-H]^-$	2
2163	3.56	PC(16:0_20:5)	$[M+H_2CO_2-H]^-$	2
2225	5.32	PC(17:0_18:2)	$[M+CH_3COOH-H]^-$	2
4126	4.56	PC(18:0_20:4)	$[2M+H_2CO_2-H]^-$	2
2725	5.09	PC(18:0_22:4)	$[M+CH_3COOH-H]^-$	2
2626	5.20	PC(20:0_20:4)	$[M+H_2CO_2-H]^-$	2
1876	5.18	PC(O-16:0_16:0)	$[M+CH_3COOH-H]^-$	2
1849	2.05	PC(O-17:1_15:1) and PC(O-16:1_18:1)	$[M+CH_3COOH-H]^-$	2
2038	2.37	PC(O-16:0_18:1)	$[M+CH_3COOH-H]^-$	2
2205	4.51	PC(O-18:1_18:2)	$[M+CH_3COOH-H]^-$	2
2187	4.50	PC(O-16:1_20:3)	$[M+CH_3COOH-H]^-$	2
2432	5.33	PC(O-18:0_20:3)	$[M+CH_3COOH-H]^-$	2
2411	4.52	PC(O-18:1_20:3)	$[M+CH_3COOH-H]^-$	2
2412	5.04	PC(O-18:1_20:3)	$[M+CH_3COOH-H]^-$	2
2400	4.52	PC(O-18:1_20:4) and PC(O-16:0_22:5)	$[M+CH_3COOH-H]^-$	2
2587	4.33	PC(O-18:1_22:6) and PC(O-22:7_18:0)	$[M+CH_3COOH-H]^-$	2
1650	4.36	PE(16:0_20:4)	$[M-H]^-$	2

1651	4.17	PE(16:0_20:4)	$[M-H]^-$	2
1623	4.35	PE(O-15:1_22:5)	$[M-H]^-$	2
1699	4.33	PE(O-18:3_20:4)	$[M-H]^-$	2
1641	4.38	PE(O-22:8_18:0) and PE(O-18:2_22:6)	$[M-H]^-$	2
1837	4.36	PE(O-22:8_18:0) and PE(O-18:2_22:6)	$[M-H]^-$	2
2631	3.50	PI(18:1_20:4)	$[M-H]^-$	2
349	2.38	Prostaglandin A1 ethyl ester	$[M-H]^-$	3
1912	4.00	SM(d36:2)	$[M+CH_3COOH-H]^-$	2
2439	6.14	SM(d41:2)	$[M+CH_3COOH-H]^-$	2
2557	7.17	SM(d42:1)	$[M+CH_3COOH-H]^-$	2
78	2.21	FA(17:1)	$[M-H]^-$	2
886	2.27	LPE(20:0)	$[M-H]^-$	2
5443	9.32	DG(40:0)	$[M+NH_4]^+$	2
10366	7.56	TG(60:12)	$[M+NH_4]^+$	2
5438	9.29	Campesterol Ester(18:2)	$[M+NH_4]^+$	2
5439	9.35	Campesterol Ester(18:2)	$[M+NH_4]^+$	2
5344	6.87	Cer(d18:1_24:1)	$[M+H]^+$	2
6813	6.54	HexCer(d18:1_24:1)	$[M+H]^+$	2
6573	4.80	PC(37:3)	$[M+H]^+$	2
6839	4.95	PC(38:3)	$[M+H]^+$	2
7105	5.26	PC(39:4)	$[M+H]^+$	2
7357	4.52	PC(40:5)	$[M+H]^+$	2
7604	5.05	PC(41:6)	$[M+H]^+$	2
7565	5.26	PC(41:7)	$[M+H]^+$	2
7815	4.08	PC(42:8)	$[M+H]^+$	2
5618	5.22	PC(O-32:0)	$[M+H]^+$	2
5604	4.49	PC(O-32:1)	$[M+H]^+$	2
6538	5.08	PC(O-38:4)	$[M+H]^+$	2

6539	5.09	PC(O-38:4)	$[M+H]^+$	2
6493	4.57	PC(O-38:5)	$[M+H]^+$	2
7022	5.26	PC(O-40:6)	$[M+H]^+$	2
7023	4.63	PC(O-40:6)	$[M+H]^+$	2
6983	4.06	PC(O-40:7)	$[M+H]^+$	2
6984	4.40	PC(O-40:7)	$[M+H]^+$	2
9401	7.59	TG(56:9)	$[M+NH_4]^+$	2
8964	7.46	TG(58:11)	$[2M+K]^+$	2
9614	7.46	TG(58:11)	$[2M+NH_4]^+$	2
10101	7.46	TG(58:11)	$[M+K]^+$	2
10226	7.65	TG(58:9)	$[M+NH_4]^+$	3
10227	7.69	TG(58:9)	$[M+NH_4]^+$	3
10228	7.79	TG(58:9)	$[M+NH_4]^+$	2
10230	7.99	TG(58:9)	$[M+NH_4]^+$	2
4512	9.48	cholesterol derivative	$[M+NH_4]^+$	3

Table S2. Statistically significant lipid features for the comparison between DKO and DKO control mice that were present in at least three lifetime stages. FA: Fatty acids, PC: Phosphatidylcholines, PC-O: Ether phosphatidylcholines, Cer: Ceramides, and SM: Sphingomyelins. Confidence level for metabolite annotation was assigned based on the following criteria: 1) exact mass, isotopic pattern, retention time, and MS/MS spectrum of standard matched to the feature. 2) exact mass, isotopic pattern, and MS/MS spectrum matched with in-house MS/MS library or literature spectra, or fragmentation ions observed are consistent with proposed structure. 3) tentative ID assignment based on elemental formula match with literature. 4) unknowns.

ID	Retention Time [min]	Lipids	Confidence Level
201	1.13	15-deoxy-D-12,14-Prostaglandin A2	3
1111	4.69	Cer(d33:1)	2
1454	6.64	Cer(d41:2)	2
2163	3.56	PC(16:0_20:5)	2
2409	4.77	PC(18:0_20:4)	2
2725	5.09	PC(18:0/22:4)	2
1849	2.05	PC(O-17:1_15:1) and PC(O-16:1_18:1)	2
2587	4.33	PC(O-18:1_22:6) and PC(O-22:7_18:0)	2
349	2.38	Prostaglandin A1 ethyl ester	3
2238	6.32	SM(t39:0) or SM(d39:0-OH)	2
4431	1.64	FA(18:3)	2
5618	5.22	PC(O-32:0)	2
5604	4.49	PC(O-32:1)	2
6538	5.08	PC(O-38:4)	2
6539	5.09	PC(O-38:4)	2
6493	4.57	PC(O-38:5)	2
7022	5.26	PC(O-40:6)	2
7023	4.63	PC(O-40:6)	2

6983	4.06	PC(O-40:7)	2

Table S3. **Lipids selected *via* machine learning for each percentage lifetime stage.** Confidence level for metabolite annotation was assigned based on the following criteria: 1) exact mass, isotopic pattern, retention time, and MS/MS spectrum of standard matched to the feature. 2) exact mass, isotopic pattern, and MS/MS spectrum matched with in-house MS/MS library or literature spectra, or fragmentation ions observed are consistent with proposed structure. 3) tentative ID assignment based on elemental formula match with literature. 4) unknowns.

ID	Retention Time [min]	Lipids	Confidence Level
**Lifetime Stage I: 0-30% Lifetime**			
12	1.04	3-hydroxyphenyl-valerate	3
1472	4.67	PE(O-34:3)	2
2226	4.51	PC(17:0_18:2)	2
1941	3.88	PC(38:6)	2
1560	4.16	PE(O-16:1_20:5)	2
**Lifetime Stage II: 30-45% Lifetime**			
452	4.36	FA(26:1)	2
2463	6.75	HexCer(d40:0-OH)	2
2446	6.37	HexCer(d40:1-OH)	2
1679	4.14	PC(16:0_18:2)	2
2091	3.76	PC(16:0_18:3)	2
2294	3.42	PC(18:2_18:2)	2
1789	3.66	PC(16:0_20:5)	2
2165	3.41	PC(16:1_20:4)	2
4125	4.78	PC(18:0_20:4)	2
2143	4.09	PC(18:1_20:4) and PC(16:0_22:5)	2
2725	5.09	PC(18:0_22:4)	2
1876	5.18	PC(O-16:0_16:0)	2
2207	4.65	PC(O-18:1_18:2)	2
2187	4.50	PC(O-16:1_20:3)	2

2411	4.52	PC(O-18:1_20:3)	2
2412	5.04	PC(O-18:1_20:3)	2
2384	3.98	PC(O-16:1_22:5)	2
2587	4.33	PC(O-18:1_22:6) and PC(O-22:7_18:0)	2
1859	4.35	PE(17:0_22:6)	2
1765	3.42	SM(d34:2)	2
2540	6.23	SM(d42:2)	2
2541	6.50	SM(d42:2)	2
4431	1.64	FA(18:3)	2
6573	4.80	PC(37:3)	2
7022	5.26	PC(O-40:6)	2
**Lifetime Stage III: 45-60% Lifetime**			
1111	4.70	Cer(d33:1)	2
1454	6.64	Cer(d41:2)	2
1726	4.82	Cer(d45:1)	2
2246	6.12	HexCer(d38:0-OH)	2
2773	7.14	HexCer(d40:0) or HexCer(t42:0-OH)	2
2265	5.90	PC(18:0_18:0)	2
2443	4.04	PC(16:0_20:4)	2
2409	4.77	PC(18:0_20:4)	2
2725	5.09	PC(18:0_22:4)	2
1608	5.64	PE(O-18:0_18:2)	2
2629	3.56	PI(18:1_20:4)	2
2631	3.50	PI(18:1_20:4)	2
349	2.38	Prostaglandin A1 ethyl ester	3
4450	2.54	6-methyl-1-(2-methylphenyl)-3-propylfuro[3,2-d]pyrimidine-2,4(1H,3H)-dione	3
4434	1.89	FA(18:2)	2
6433	4.11	PC(37:6)	2
6445	4.44	PC(O-38:6)	2

8429	8.52	TG(18:0_18:1_18:2)_and_TG(18:1_18:1_18:1)_and_TG(16:0_18:2_20:1)	2
**Lifetime Stage IV: 60-75% Lifetime**			
277	1.32	FA(20:1-2OH)	2
229	1.66	FA(20:1-OH)	2
270	1.69	FA(20:2-2OH)	2
1111	4.69	Cer(d33:1)	2
1726	4.82	Cer(d45:1)	2
1762	4.32	HexCer(d34:1)	2
2725	5.09	PC(18:0_22:4)	2
2704	4.81	PC(18:0_22:5)	2
1727	4.36	PC(O-15:1_20:4)	2
2587	4.33	PC(O-18:1_22:6) and PC(O-22:7_18:0)	2
2630	3.35	PI(18:1_20:4)	2
2439	6.14	SM(d41:2)	2
2238	6.32	SM(t39:0) or SM(d39:0-OH)	2
6241	4.40	PC(O-37:5)	2
6573	4.80	PC(37:3)	2
6156	5.62	PC(37:4)	2
6435	4.10	PC(37:6)	2
7103	4.73	PC(39:4)	2
5618	5.22	PC(O-32:0)	2
6538	5.08	PC(O-38:4)	2
6539	5.09	PC(O-38:4)	2
6983	4.06	PC(O-40:7)	2
5593	4.32	SM(d35:1)	2
8964	7.46	TG(58:11)	2
**Lifetime Stage V: 75-100% Lifetime**			
772	1.65	LPE(18:1)	2
1874	5.52	PE(P-40:4) or PE(O-40:5)	3

1297	6.79	Cer(d42:2)	2
1473	4.33	HexCer(d34:1)	2
934	1.74	LPC(20:4_0:0)_and_LPC(0:0_20:4)	2
1122	1.50	LPC(20:5_0:0)_and_LPC(0:0_20:5)	2
357	3.63	FA(24:1)	2
1790	3.95	PC(14:0_16:0)	2
2412	5.04	PC(O-18:1_20:3)	2
2384	3.98	PC(O-16:1_22:5)	2
2619	6.05	PC(O-18:1_22:4)	2
2587	4.33	PC(O-18:1_22:6) and PC(O-22:7_18:0)	2
2706	4.48	PC(O-18:1_22:6) and PC(O-22:7_18:0)	2
2707	4.33	PC(O-18:1_22:6) and PC(O-22:7_18:0)	2
1884	6.07	PE(O-18:0_22:4); PE(O-40:4)	2
2063	6.68	PE(O-18:2_24:2)	2
2021	5.98	PE(O-20:1_22:6)	2
2436	3.31	PI(16:0_20:4)	2
1912	4.00	SM(d36:2)	2
2029	5.03	SM(d37:1)	2
2116	5.45	SM(d38:1)	2
2454	6.62	SM(d41:1)	2
9666	8.02	TG(58:10)	3
9784	8.24	TG(58:8)	3
10458	8.55	TG(60:10)	3
10366	7.56	TG(60:12)	2
6612	4.57	PC(37:2)	3
6839	4.95	PC(38:3)	2
7402	5.72	PC(40:4)	2
7357	4.52	PC(40:5)	2
7915	4.65	PC(42:6)	3
6539	5.09	PC(O-38:4)	2

6493	4.57	PC(O-38:5)	2
6983	4.06	PC(O-40:7)	2
5652	4.08	SM(d36:2)	2
5891	4.69	SM(d38:4)	2
7197	8.37	TG(16:0_16:0_18:1)	2
10287	8.18	TG(18:0_20:4_20:4) and TG(18:0_18:2_22:6)	2
10226	7.65	TG(58:9)	3
10227	7.69	TG(58:9)	3
10230	7.99	TG(58:9)	2
4512	9.48	cholesterol derivative	3

Table S4. Machine learning results for DKO classification. k-NN: k-Nearest Neighbors, RF: Random Forests, SVM: Support Vector Machine, Voting: Voting Classifier. CV: cross-validation. All scores are ROC AUC.

Machine learning algorithm	Training set CV scores	Test set scores
**Lifetime Stage I: 0-30% Lifetime**		
Logistic regression	0.78(±0.16)	0.74
RF	0.82(±0.17)	0.80
k-NN	0.77(±0.24)	0.80
SVM	0.73(±0.19)	0.74
Voting	0.76(±0.20)	0.80
**Lifetime Stage II: 30-45% Lifetime**		
Logistic regression	0.76(±0.21)	0.66
RF	0.87(±0.09)	0.70
k-NN	0.79(±0.12)	0.66
SVM	0.80(±0.11)	0.62
Voting	0.82(±0.13)	0.66
**Lifetime Stage III: 45-60% Lifetime**		
Logistic regression	0.66(±0.08)	0.85
RF	0.76(±0.13)	0.77
k-NN	0.81(±0.09)	0.80
SVM	0.80(±0.06)	0.78
Voting	0.77(±0.09)	0.82
**Lifetime Stage IV: 60-75% Lifetime**		
Logistic regression	0.80(±0.08)	0.47
RF	0.83(±0.13)	0.66
k-NN	0.76(±0.14)	0.54
SVM	0.78(±0.07)	0.57
Voting	0.82(±0.11)	0.54
**Lifetime Stage V: 75-100% Lifetime**		

Logistic regression	0.90(±0.06)	0.69
RF	0.90(±0.04)	0.63
k-NN	0.90(±0.06)	0.67
SVM	0.90(±0.05)	0.75
Voting	0.91(±0.04)	0.74

Table S5. Composition of stable isotope-labeled chemical standards mixture used in UHPLC-MS.

**Isotopically labeled lipids**	**CAS number**	**Concentration in stock solution** (mg/ml)
LPC (18:1(d7))	2097561-13-0	25
LPE(18:1(d7)	2260669-47-2	5
PC (15:0/18:1(d7))	2097561-16-3	160
PE (15:0/18:1(d7))	2097561-15-2	5
PS (15:0/18:1(d7))	2260669-40-5	10
PG (15:0/18:1(d7))	2260669-42-7	30
PI (15:0/18:1(d7))	2260669-44-9	20
CE (18:1(d7))	1416275-35-8	350
DG (15:0/18:1(d7))	2097561-14-1	10
TG (15:0/18:1(d7)/15:0)	2097561-17-4	55
SM (18:1(d9))	2260669-50-7	30
cholesterol-d7	83199-47-7	100

Table S6. Chromatographic gradient for RP UHPLC-MS method. For negative ion mode, mobile phase A was 10 mM ammonium acetate with water/acetonitrile (40:60 v/v) and mobile phase B was 10 mM ammonium acetate with 2-isopropanol/acetonitrile (90:10 v/v). For positive ion mode, mobile phase A was 10 mM ammonium formate with water/acetonitrile (40:60 v/v) and 0.1% formic acid. Mobile phase B was 10 mM ammonium formate with 2-isopropanol/acetonitrile (90:10 v/v) and 0.1% formic acid.

**RP UHPLC Gradient**			
**Time (min)**	**Mobile phase A**	**Mobile phase B**	**Flow rate (ml min^{-1})**
0.0	80%	20%	0.4
0.0	80%	20%	0.4
1.0	40%	60%	0.4
5.0	30%	70%	0.4
5.5	15%	85%	0.4
8.0	10%	90%	0.4
8.2	0%	100%	0.4
10.5	0%	100%	0.4
10.7	80%	20%	0.4
12.0	80%	20%	0.4

Table S7. MS parameters used for RP UHPLC-MS. Arb: Arbitrary units.

**MS parameters for RP UHPLC-MS**		
**MS parameter**	**Positive mode**	**Negative mode**
Capillary temperature	275 °C	275 °C
Spray voltage	+ 3.5kV	- 2.5kV
Sheath gas flow rate	40 Arb.	40 Arb.
Auxiliary gas flow rate	8 Arb.	8 Arb.
Sweep gas flow rates	1 Arb.	1 Arb.
Vaporizer temperature	320 °C	320 °C
